# The effect of immunonutrition on postoperative ileus following colorectal cancer surgery: a systematic review and meta-analysis of randomized controlled trials

**DOI:** 10.3389/fnut.2026.1778464

**Published:** 2026-04-10

**Authors:** Yuqiang Zhang, Chang Chen, Bo Dong, Guanglin Li

**Affiliations:** Department of General Surgery, People's Hospital of Rongchang District, Chongqing, China

**Keywords:** colorectal cancer, gastrointestinal function, immunonutrition, meta-analysis, postoperative ileus

## Abstract

**Objective:**

The effect of immunonutrition on postoperative gastrointestinal recovery in patients undergoing colorectal cancer surgery remains controversial. We conducted a systematic review and meta-analysis of randomized controlled trials (RCTs) to evaluate the efficacy of immunonutrition in alleviating postoperative ileus following colorectal cancer surgery.

**Methods:**

We searched PubMed, Web of Science, Embase, China National Knowledge Infrastructure, VIP, and Cochrane Library databases for eligible studies. Risk ratios (RRs) and mean differences (MDs) with 95% confidence intervals (CIs) were calculated.

**Results:**

A total of 28 RCTs involving 2,367 patients were included. Compared with the control group, immunonutrition supplementation significantly shortened the time to first flatus (MD, −0.56 days; 95% CI, −0.74, −0.39, *p* < 0.00001), time to first defecation (MD, −0.51 days; 95% CI, −0.86, −0.15, *p* = 0.005), and postoperative length of hospital stay (MD, −1.47 days; 95% CI, −2.39, −0.55, *p* = 0.002). Moreover, immunonutrition reduced the incidence of overall postoperative complications (RR 0.64, 95% CI 0.55, 0.75) and postoperative abdominal distension (RR 0.33, 95% CI 0.12, 0.89).

**Conclusion:**

Perioperative immunonutrition supplementation significantly shortened the time to first flatus and time to first defecation after colorectal cancer surgery. Further large-scale, multicenter RCTs are warranted to confirm these findings and establish optimal supplementation protocols.

**Systematic review registration:**

https://www.crd.york.ac.uk/PROSPERO/view/CRD420251250520, CRD420251250520.

## 1 Introduction

Colorectal cancer is one of the most prevalent malignancies worldwide, accounting for more than 900,000 cancer-related deaths annually (1). Surgical resection remains the primary treatment modality (2). However, postoperative ileus is a frequent complication following colorectal cancer surgery, with an incidence of approximately 10–20% (3). Postoperative ileus is characterized by a transient impairment of bowel motility, clinically manifested as abdominal distension, abdominal pain, nausea, vomiting, and delayed passage of flatus and stool (4). This condition not only prolongs hospital stay but also increases postoperative morbidity and imposes a substantial economic burden (5). In the United States alone, the annual healthcare cost associated with postoperative ileus is estimated to exceed 1.4 billion dollars (6). Therefore, identifying effective strategies to improve postoperative ileus in patients undergoing colorectal cancer surgery is of considerable clinical significance.

Immunonutrition refers to nutritional supplements or interventions that beneficially modulate the immune system (7). Experimental studies have demonstrated that immunonutrition can protect the intestinal mucosa and reduce mucosal injury in animal models (8–10). Several clinical trials have also investigated the impact of immunonutrition on postoperative ileus in colorectal cancer patients; however, the results remain inconsistent (11–15). Han et al. reported that preoperative immunonutrition significantly shortened the time to first flatus, time to first defecation, and length of hospital stay (14). In contrast, Pannirselvam et al. found no significant benefit of immunonutrition on postoperative bowel function recovery (15).

Given these conflicting results, high-quality evidence is needed to clarify whether immunonutrition can effectively prevent postoperative ileus. Therefore, we systematically identified all available randomized controlled trials (RCTs) and performed a meta-analysis to evaluate the effects of immunonutrition on postoperative gastrointestinal recovery in patients undergoing colorectal cancer surgery.

## 2 Methods

### 2.1 Search strategy

This review followed the Preferred Reporting Items for Systematic Reviews and Meta-Analyses (PRISMA) guidelines. The protocol was registered in PROSPERO (CRD420251250520). PubMed, Web of Science, Embase, China National Knowledge Infrastructure, VIP, and Cochrane Library databases were searched from inception through September 28, 2025. The complete search strategy is presented in Table 1. The literature screening was conducted independently by two authors (Yuqiang Zhang and Bo Dong). Any disagreements were resolved through discussion with a third author (Chang Chen). Furthermore, we manually retrieved the reference lists of the eligible studies to identify other relevant studies. The retrieval process had no language restrictions.

**Table 1:** Electronic search strategy.

Database	Search term (published up to September 28, 2025)	Number
Web of science	((TS = (colorectal cancer OR colorectal tumor OR colorectal neoplasm OR colorectal carcinoma OR colorectal malignancies OR rectal cancer OR rectal malignancies OR rectal tumor OR rectal neoplasm OR rectal carcinoma OR colon cancer OR colon malignancies OR colon tumor OR colon neoplasm OR colon carcinoma OR colon malignancies) AND (TS = (immunonutrition OR immune nutrition OR immune-enhanced nutrition OR arginine OR glutamine OR omega 3 fatty acid))) AND TS = (randomized controlled trial OR randomized OR clinical trial OR RCT)	370
Embase	(colorectal cancer OR colorectal tumor OR colorectal neoplasm OR colorectal carcinoma OR colorectal malignancies OR rectal cancer OR rectal malignancies OR rectal tumor OR rectal neoplasm OR rectal carcinoma OR colon cancer OR colon malignancies OR colon tumor OR colon neoplasm OR colon carcinoma OR colon malignancies).ab,kw,ti. AND (immunonutrition OR immune nutrition OR immune-enhanced nutrition OR arginine OR glutamine OR omega 3 fatty acid).ab,kw,ti. AND (randomized controlled trial OR randomized OR clinical trial OR RCT).ab,kw,ti.	135
PubMed	(colorectal cancer[Title/Abstract] OR colorectal tumor[Title/Abstract] OR colorectal neoplasm[Title/Abstract] OR colorectal carcinoma[Title/Abstract] OR colorectal malignancies[Title/Abstract] OR rectal cancer[Title/Abstract] OR rectal malignancies[Title/Abstract] OR rectal tumor[Title/Abstract] OR rectal neoplasm[Title/Abstract] OR rectal carcinoma[Title/Abstract] OR colon cancer[Title/Abstract] OR colon malignancies[Title/Abstract] OR colon tumor[Title/Abstract] OR colon neoplasm[Title/Abstract] OR colon carcinoma[Title/Abstract] OR colon malignancies[Title/Abstract]) AND (immunonutrition[Title/Abstract] OR immune nutrition[Title/Abstract] OR immune-enhanced nutrition[Title/Abstract] OR arginine[Title/Abstract] OR glutamine[Title/Abstract] OR omega 3 fatty acid[Title/Abstract]) AND (randomized controlled trial[Title/Abstract] OR randomized[Title/Abstract] OR clinical trial[Title/Abstract] OR RCT[Title/Abstract])	433
VIP	M = (colorectal cancer OR colorectal tumor OR colorectal neoplasm OR colorectal carcinoma OR colorectal malignancies OR rectal cancer OR rectal malignancies OR rectal tumor OR rectal neoplasm OR rectal carcinoma OR colon cancer OR colon malignancies OR colon tumor OR colon neoplasm OR colon carcinoma OR colon malignancies) AND M = (immunonutrition OR immune nutrition OR immune-enhanced nutrition OR arginine OR glutamine OR omega 3 fatty acid) AND M = (randomized controlled trial OR randomized OR clinical trial OR RCT)	182
CNKI	(SU = ‘colorectal cancer’ OR SU = ‘colorectal tumor’ OR SU = ‘colorectal neoplasm’ OR SU = ‘colorectal carcinoma’ OR SU = ‘colorectal malignancies’ OR SU = ‘rectal cancer’ OR SU = ‘rectal malignancies’ OR SU = ‘rectal tumor’ OR SU = ‘rectal neoplasm’ OR SU = ‘rectal carcinoma’ OR SU = ‘colon cancer’ OR SU = ‘colon malignancies’ OR SU = ‘colon tumor’ OR SU = ‘colon neoplasm’ OR SU = ‘colon carcinoma’ OR SU = ‘colon malignancies’) AND (SU = ‘immunonutrition’ OR SU = ‘immune nutrition’ OR SU = ‘immune-enhanced nutrition’ OR SU = ‘arginine’ OR SU = ‘glutamine’ OR SU = ‘omega 3 fatty acid’)	293
Cochrane library trials	((colorectal cancer OR colorectal tumor OR colorectal neoplasm OR colorectal carcinoma OR colorectal malignancies OR rectal cancer OR rectal malignancies OR rectal tumor OR rectal neoplasm OR rectal carcinoma OR colon cancer OR colon malignancies OR colon tumor OR colon neoplasm OR colon carcinoma OR colon malignancies):ti,ab,kw) AND ((immunonutrition OR immune nutrition OR immune-enhanced nutrition OR arginine OR glutamine OR omega 3 fatty acid):ti,ab,kw) AND ((randomized controlled trial OR randomized OR clinical trial OR RCT):ti,ab,kw)	274

### 2.2 Study selection

Studies were chosen based on the PICO criteria (Patient, Intervention, Comparison, Outcomes, Study Type), as shown below: (a) Patient: Patients undergoing colorectal cancer surgery; (b) Intervention: patients received immunonutrition support (including arginine, n-3 omega fatty acids, or glutamine); (c) Comparison: patients received a standard diet or placebo; (d) Outcomes: outcome measured at least one of primary outcomes. Primary outcomes encompassed time to first flatus, time to first defecation, and postoperative ileus. Secondary outcomes included length of hospital stay, overall postoperative complications, and abdominal distension; (e) Study type: RCTs.

Exclusion criteria included: case reports, non-RCTs, conference abstracts, animal studies, studies without an interest outcome indicator, and letters.

### 2.3 Data extraction

Data from all eligible studies were independently extracted by two authors (Yuqiang Zhang and Bo Dong) based on a previously established form, and any disagreements were resolved by discussion with a third-party independent author (Chang Chen). The information recorded included author name, year of publication, study design, country, study population (sample size and diagnosis), type of intervention and outcome information (time to first flatus, time to first defecation, postoperative ileus, length of hospital stay, overall postoperative complications, and abdominal distension). When data of interest cannot be obtained from the article, the corresponding author was contacted to obtain the necessary data.

### 2.4 Quality assessment

The quality assessment was conducted independently by two authors (Yuqiang Zhang and Bo Dong) using the Cochrane risk-of-bias tool 2: (a) randomization process, (b) deviations from intended interventions, (c) missing outcome data, (d) measurement of the outcome, (e) selection of the reported results, and (f) overall risk of bias. Any disagreements were resolved through discussion by a third author (Chang Chen).

### 2.5 Statistical analysis

The meta-analysis was performed using the Revman 5.3 (The Nordic Cochrane Centre, The Cochrane Collaboration 2014; Copenhagen, Denmark) and Stata 14 software. Risk ratios (RRs) with corresponding 95% confidence intervals (CI) were calculated for categorical outcome variables (postoperative ileus, overall postoperative complications, and abdominal distension) and mean difference (MD) for continuous outcome variables (time to first flatus, time to first defecation, and length of hospital stay). Heterogeneity across studies was assessed by using the Q test and I^2^ test. A random-effects model was used if I^2^ > 50%; otherwise, a fixed-effects model was used (16). The sensitivity analysis was conducted using one-study exclusion method to evaluate the impact of each study on the total effect size. Potential publication bias was assessed using Egger’s test and funnel plot (if the number of studies exceeds 10). Statistical significance was established at *p* < 0.05.

## 3 Results

### 3.1 Literature retrieval

**Figure 1:**
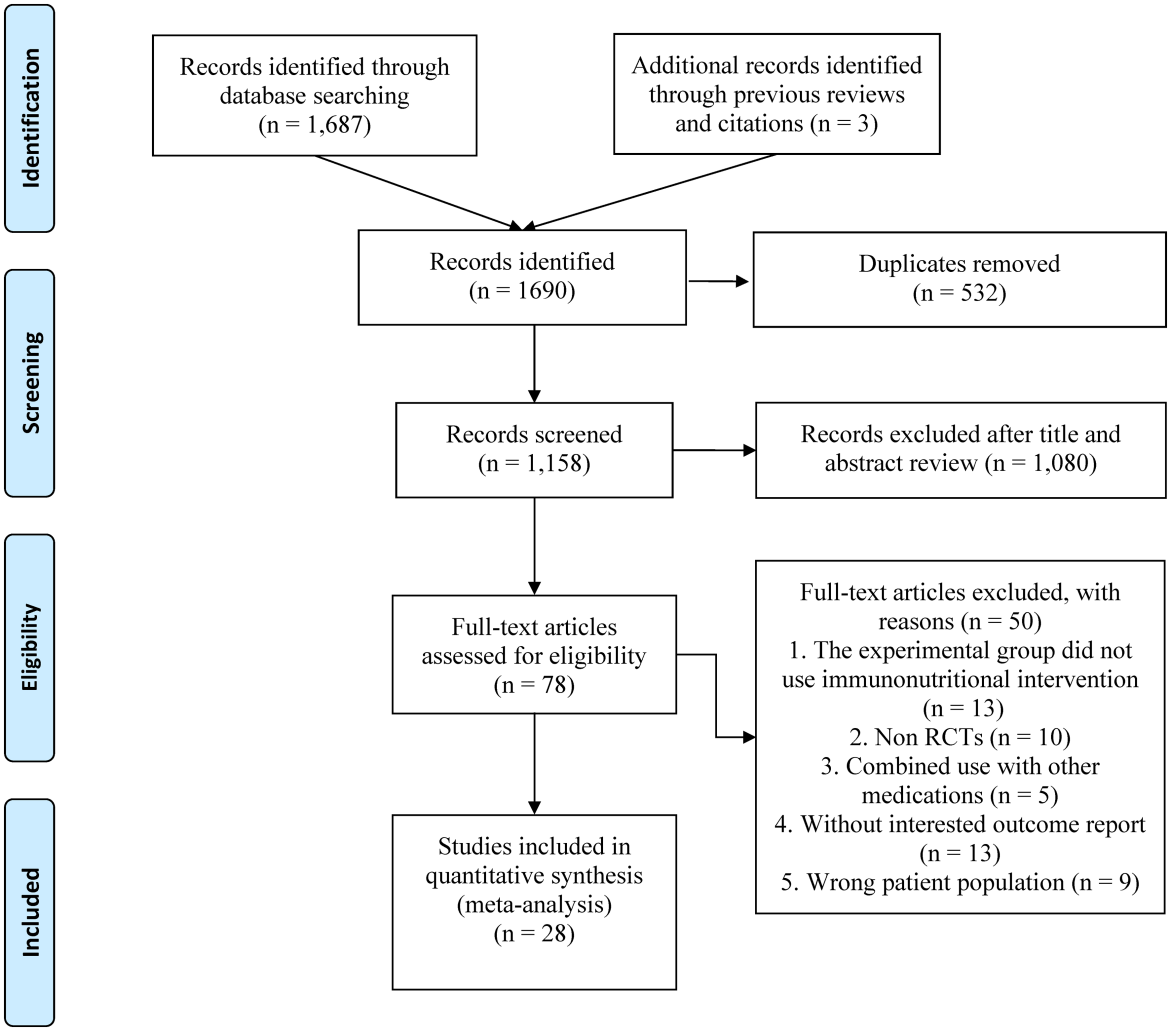
The PRISMA flowchart.

The initial database query retrieved 1,690 records (Figure 1), of which 532 were duplicates. After reviewing titles and abstracts, 1,080 papers were excluded, and the full texts of the remaining 78 studies were evaluated. Finally, 28 eligible studies (7, 11–15, 17–38) were enrolled in this study.

### 3.2 Study characteristics and quality assessment

**Figure 2:**
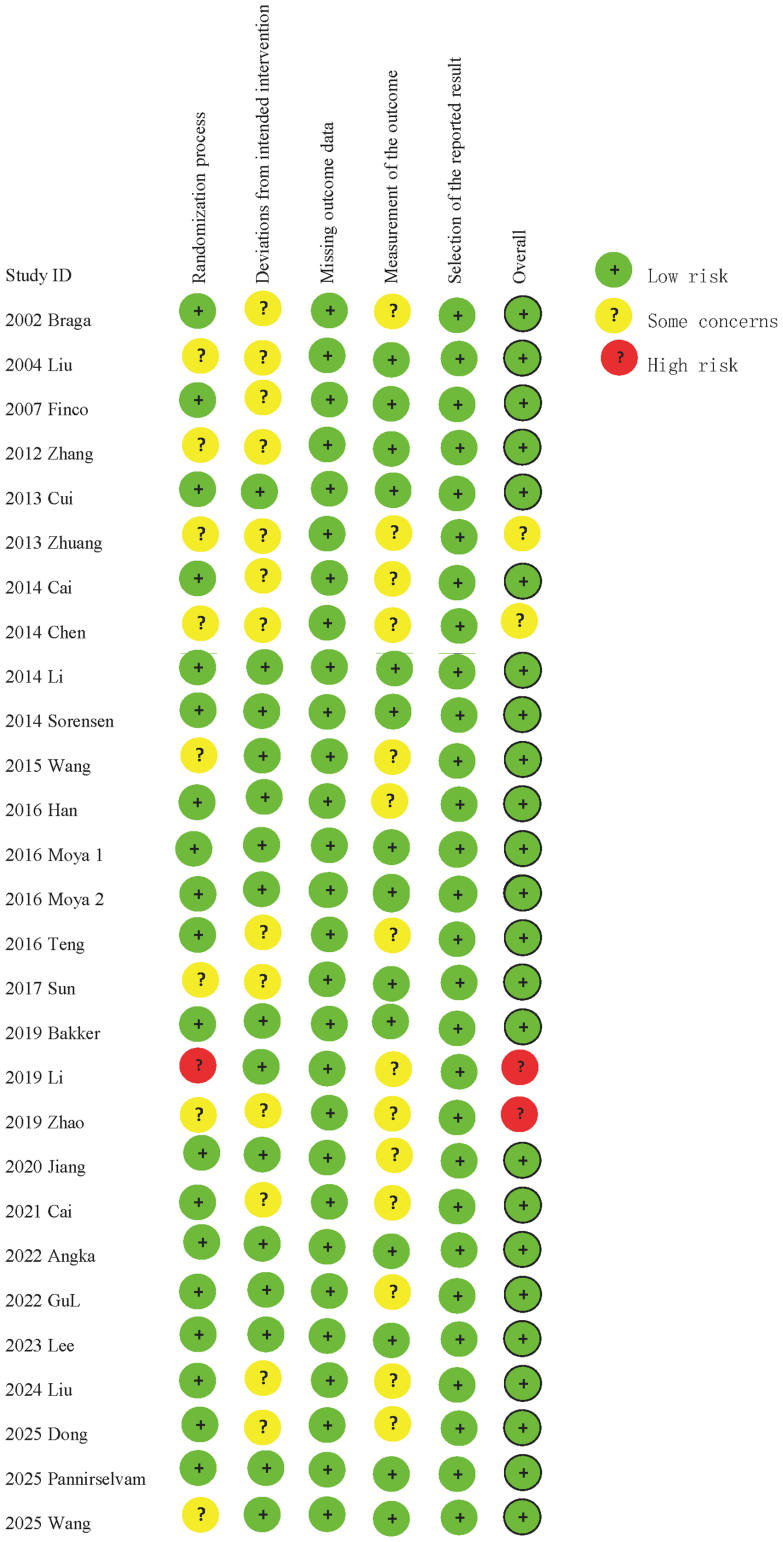
Risk of bias for each included study.

The studies were published between 2002 and 2025 and included 2,367 patients (immunonutrition group: 1,179 patients; control group: 1,188 patients). The indications for surgical treatment were only for colon cancer or rectal cancer in 5 studies, while 23 studies included patients with both colon cancer and rectal cancer. The included patients were mainly from South Korea, Italy, Japan, United Kingdom, Spain, China, Netherlands, Canada, Malaysia, and Turkey. The detailed characteristics of the 28 studies (7, 11–15, 17–38) are presented in Table 2. In quality assessment, 24 studies were assessed as being of low risk of bias. 19 studies used appropriate randomization methods, 6 studies were double-blind, and appropriate allocation concealment was conducted. The risk of selection bias in all studies was assessed as low risk (Figure 2).

**Table 2:** Study characteristics of the 28 included studies.

First author, year	Design	Period of study	Tumor types	Sample size	Sex (male)	Ages (year)	Immunonutrition protocol
Braga et al. (30)	RCT	2002	Colorectal cancer	Immunonutrition: 50 Control: 50	Immunonutrition: 30 Control: 29	Immunonutrition: 63 (8.1) Control: 62.2 (10.4)	Drink 1 L/day of a liquid diet supplemented with arginine (12.5 g/L) and n-3 fatty acids (3.3 g/L), (Oral Impact; Novartis, Bern, Switzerland) for 5 days before operation
Liu et al. (13)	RCT	2001–2003	Colorectal cancer	Immunonutrition: 20 Control: 20	Immunonutrition: NA Control: NA	Immunonutrition: NA Control: NA	Starting 3 days before the surgery, take two bottles of Rui Neng orally daily
Finco et al. (31)	RCT	2004–20,005	Colorectal cancer	Immunonutrition:14 Control: 14	Immunonutrition: 10 Control: 10	Immunonutrition: 66.1 (11.2) Control: 68.1 (12.9)	During the 6 days before surgery, these patients were given 750 mL/day of a diet enriched with arginine, omega-3 fatty acids, and RNA (Oral Impact; Gland, Swiss) associated with a low-fiber diet. Beginning on postoperative day 1, they were fed with enteral immunonutrients for 3 days, then with a conventional diet.
Zhang (12)	RCT	2010–2012	Colorectal cancer	Immunonutrition:20 Control: 20	Immunonutrition: NA Control: NA	Immunonutrition: NA Control: NA	Starting 1 day after surgery, supplement with 30 g/d glutamine
Cui et al. (32)	RCT	2011	Colon cancer	Immunonutrition: 20 Control: 20	Immunonutrition: 12 Control: 10	Immunonutrition: 55 (10.8) Control: 59 (11.9)	Intravenous infusion of 3.4% alanyl-L- glutamine (Sino-Swed Pharmaceutical Corp. Ltd). The total volume of each solution was 22.4 mL/kg and it was administered 24 h before and 1 h after the start of surgery. The stock solution of glutamine (20%) was diluted to 3.4% with 8.5% 18AA-II for intravenous infusion.
Zhuang et al. (11)	RCT	2010–2012	Colorectal cancer	Immunonutrition: 20 Control: 20	Immunonutrition: NA Control: NA	Immunonutrition: NA Control: NA	Starting from the first day after surgery, Rui Neng was supplemented at a rate of 20–30 mL/h. On the second day after surgery, it increased to 30–40 mL/h gradually, and on the third to seventh day, it was 50–70 mL/h
Cai et al. (29)	RCT	2010–2012	Colorectal cancer	Immunonutrition: 20 Control: 20	Immunonutrition: NA Control: NA	Immunonutrition: NA Control: NA	On the first day after surgery, 1,000 mL of Rui neng (containing 3 g of omega-3 polyunsaturated fatty acids) was infused at a rate of 10 mL/h to 20 mL/h. On the second day after surgery, the infusion rate increased to 20 mL/h to 30 mL/h, gradually increasing, and on the third to seventh day, it increased to 30 mL/h to 50 mL/h
Chen (28)	RCT	2014	Colorectal cancer	Immunonutrition: 22 Control: 22	Immunonutrition: NA Control: NA	Immunonutrition: NA Control: NA	Starting from 1 day after surgery, supplement with 30 g/day of glutamine granules
Li et al. (27)	RCT	2012–2013	Colorectal cancer	Immunonutrition: 58 Control: 65	Immunonutrition: 37 Control: 30	Immunonutrition: 58.44 (10.96) Control: 58.62 (11.39)	Starting from 3 days before surgery, use an immune enhanced EN preparation (1,000 mL of Rui Neng containing 3 g of omega-3 polyunsaturated fatty acids)
Sorensen et al. (33)	RCT	2007–2010	Colorectal cancer	Immunonutrition: 74 Control: 74	Immunonutrition: 44 Control: 36	Immunonutrition: 69 (11) Control: 71 (10)	Received the omega-3 fatty acids (3 g twice daily, once in the morning and once in the afternoon) for 7 days before and 7 days after surgery.
Wang et al. (10)	RCT	2015	Colorectal cancer	Immunonutrition: 35 Control: 35	Immunonutrition: 21 Control: 23	Immunonutrition: 72.36 (3.36) Control: 62.84 (3.11)	Starting 2 days after surgery, oral or nasal feeding of immune enhanced nutrition 1,000 mL/d (1,000 mL of immune nutrition containing 13.0 g of glutamine, 75 g of protein, 12.5 g of L-arginine, and 2.8 g of omega-3 fatty acids)
Han et al. (14)	RCT	2011–2014	Rectal cancer	Immunonutrition: 56 Control: 56	Immunonutrition: NA Control: NA	Immunonutrition: NA Control: NA	Starting from 1 day after surgery, use the enteral immunonutritional preparation Renen (mainly containing glutamine, arginine, and omega-3 polyunsaturated fatty acids)
Moya et al. (37)	RCT	2012–2014	Colorectal cancer	Immunonutrition: 61 Control: 61	Immunonutrition: 30 Control: 27	Immunonutrition: 68 (45–92) Control: 69 (51–85)	Two cartons (400 mL) of their assigned supplement per day for 7 days prior to surgery and to keep daily records of the volume
Moya et al. (38)	RCT	2014–2015	Colorectal cancer	Immunonutrition: 121 Control: 121	Immunonutrition: 62 Control: 69	Immunonutrition: 70 (42–88) Control: 68 (41–89)	2 cartons (400 mL) of their assigned feed per day for 7 days prior to surgery and to daily record the volume consumed
Teng et al. (25)	RCT	2014–2016	Colorectal cancer	Immunonutrition: 40 Control: 40	Immunonutrition: NA Control: NA	Immunonutrition: NA Control: NA	Starting from the first day after surgery, use 100 mL/day of 10% fish oil fat emulsion
Sun et al. (24)	RCT	2014–2015	Colorectal cancer	Immunonutrition: 48 Control: 48	Immunonutrition: 30 Control: 35	Immunonutrition: 60.1 (5.7) Control: 61.7 (6.5)	Starting from the third day after surgery, use fish oil 0.2 g/ (kg. d)
Bakker et al. (36)	RCT	2014–2016	Colon cancer	Immunonutrition: 18 Control: 23	Immunonutrition: 13 Control: 15	Immunonutrition: 65(64–75) Control: 69 (63–74)	0.2 g fish oil/kg–1d–1 during 4 h between 18:00 and 22:00 the evening before surgery and another infusion the morning after surgery between 08:00 and 12:00
Li and Shiting (23)	RCT	2016–2017	Colorectal cancer	Immunonutrition: 31 Control: 31	Immunonutrition: 19 Control: 19	Immunonutrition: 62.6 (9.6) Control: 65.5(9)	Starting from 12 h after surgery, glutamine was supplemented at a rate of 0.5 g/kg · d, administered in three doses until the 7th day after surgery
Zhao (22)	RCT	2016–2017	Colorectal cancer	Immunonutrition: 32 Control: 28	Immunonutrition: 22 Control: 19	Immunonutrition: 56.75 (5.6) Control: 54.42 (5.21)	Inject 50 mL glutamine injection intravenously 24 h before surgery and 1 h after surgery, and continue for 7 days after surgery.
Jiang et al. (21)	RCT	2017–2019	Colorectal cancer	Immunonutrition: 50 Control: 50	Immunonutrition: 25 Control: 27	Immunonutrition: 61.83 (5.66) Control: 62.79 (4.87)	Administer 2 mL/kg of fish oil fat emulsion from 7 days before surgery to 7 days after surgery
Cai et al. (20)	RCT	2014–2016	Colorectal cancer	Immunonutrition: 56 Control: 56	Immunonutrition: 31 Control: 34	Immunonutrition: 67.2 (3.5) Control: 68.3 (3.6)	Starting from the 2 to 3 day after surgery, supplement glutamine 5 g/day
Angka et al. (7)	RCT	2017–2019	Colorectal cancer	Immunonutrition: 12 Control: 11	Immunonutrition: 11 Control: 8	Immunonutrition: 67.5 (23–81) Control: 65 (48–82)	From the 5 day before surgery to the 5 day after surgery, three doses per day for a total amount of 12.6 g arginine per day
Gül et al. (35)	RCT	2018–2019	Rectal cancer	Immunonutrition: 15 Control: 15	Immunonutrition: 8 Control: 9	Immunonutrition: 62.4 (2.47) Control: 57.9 (1.82)	The Immunonutrition group was given an immunonutrition (arginine, omega 3 fatty acids and nucleotides) supplement thrice daily
Lee et al. (34)	RCT	2019–2020	Colon cancer	Immunonutrition: 79 Control: 82	Immunonutrition: 56 Control: 50	Immunonutrition: 65.3 (9.2) Control: 65.3 (11.7)	400 mL/day of immunonutrient-enriched ONS (Newcare Omega, Daesang Life Science, South Korea), which contained high protein levels, arginine, and o-3 fatty acids, in addition to the normal diet for 7 consecutive days before surgery
Liu et al. (19)	RCT	2019–2022	Colorectal cancer	Immunonutrition: 75 Control: 75	Immunonutrition: 44 Control: 43	Immunonutrition: 54.39 (5.46) Control: 54.27 (5.34)	Oral immune nutrition includes substances such as arginine, glutamine, nucleotides, etc. It should be taken orally from 2–3 days before surgery and again from 6–24 h after surgery until discharge
Dong et al. (18)	RCT	2021–2023	Colorectal cancer	Immunonutrition: 41 Control: 38	Immunonutrition: 21 Control: 26	Immunonutrition: 66.34 (11.41) Control: 67.47 (9.3)	Preoperative arginine 0.25 g/once daily, orally for at least 7 days. Fish oil fat emulsion, 1 g twice a day, for at least 7 days
Pannirselvam et al. (15)	RCT	2023–2024	Colorectal cancer	Immunonutrition: 28 Control: 30	Immunonutrition: 16 Control: 20	Immunonutrition: 59.6 (13.5) Control: 60.6 (13.4)	A serving of Oral Impact consisted of 74 g of the beverage mixed with 250 mL of water. The participants consumed the Oral Impact for 7 days before their scheduled operation dates
Wang et al. (17)	RCT	2022–2024	Colorectal cancer	Immunonutrition: 63 Control: 63	Immunonutrition: 41 Control: 42	Immunonutrition: 63 (10.32) Control: 65.43 (10.5)	Starting from the second day after surgery, use Ruineng containing omega-3PUFAs

NA, not available; RCT, randomized controlled trial.

### 3.3 Meta-analysis

#### 3.3.1 Time to first flatus

**Figure 3:**
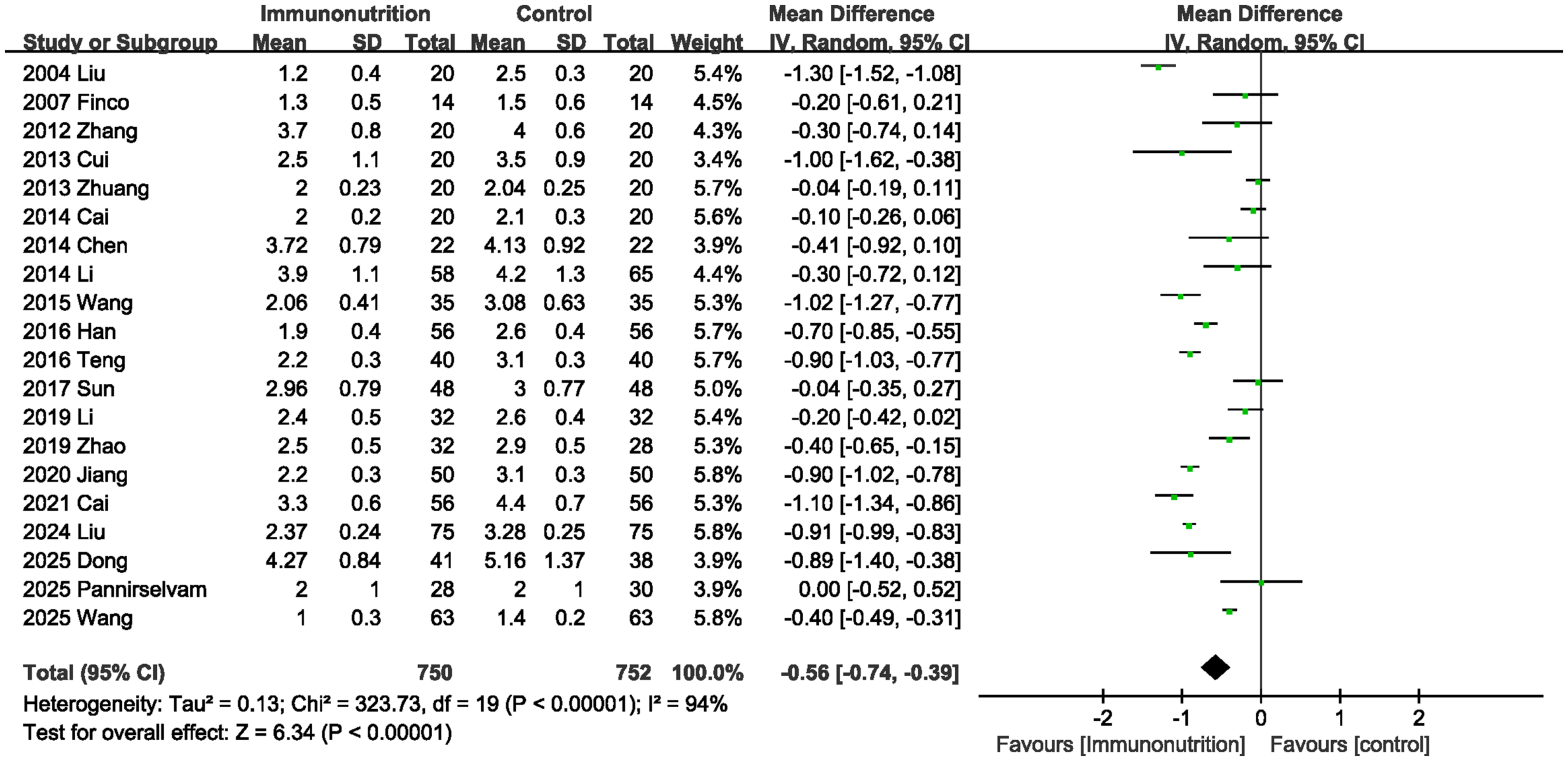
Effect of immunonutrition supplementation on time to first flatus.

Twenty studies provided information on time to first flatus. The combined results showed that perioperative supplementation of immunonutrition significantly shortened the time to first flatus, and there was significant heterogeneity between studies (MD, −0.56 days; 95% CI, −0.74, −0.39, *p* < 0.00001; I^2^ = 94%; Figure 3).

#### 3.3.2 Time to first defecation

**Figure 4:**
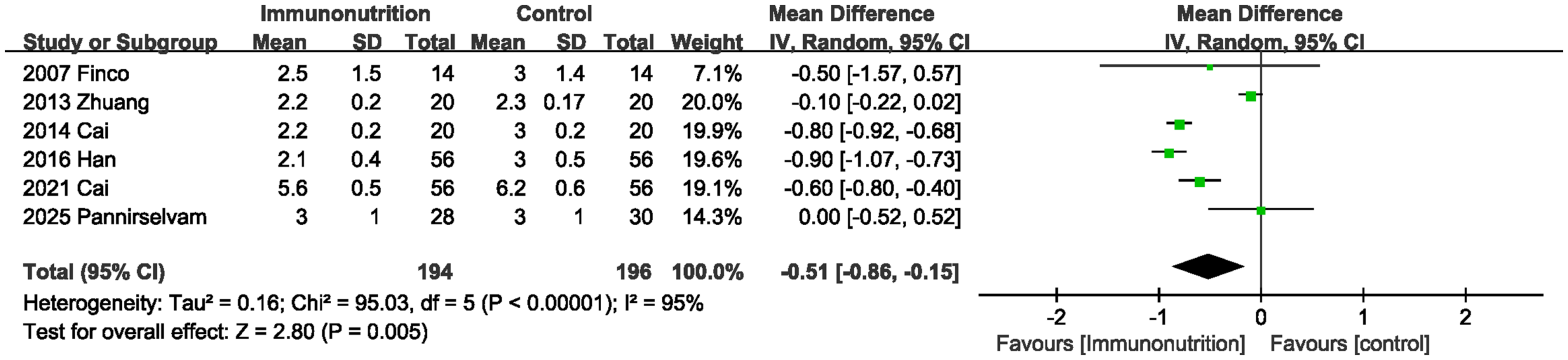
Effect of immunonutrition supplementation on time to first defecation.

The time to first defecation was reported in 6 trials. The combined results showed that supplementing with immunonutrition during the perioperative period significantly shortens the time of first defecation, and significant heterogeneity was observed between studies (MD, −0.51 days; 95% CI, −0.86, −0.15, *p* = 0.005; I^2^ = 95%; Figure 4).

#### 3.3.3 Postoperative ileus

**Figure 5:**
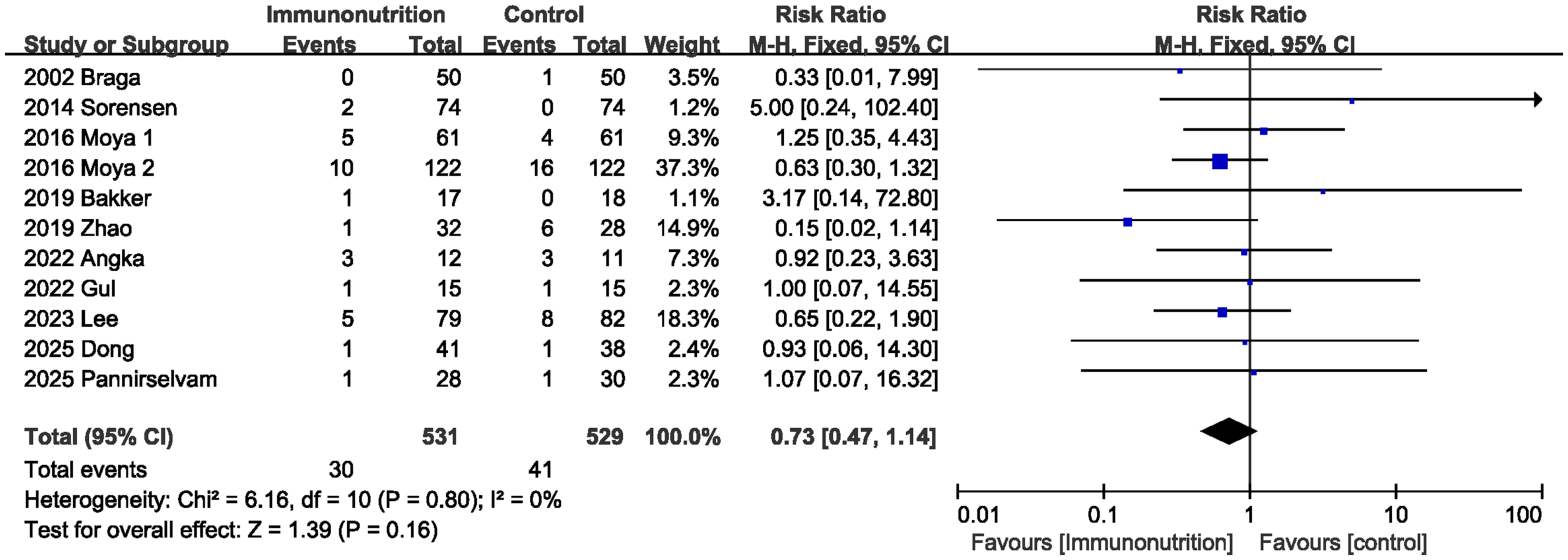
Effect of immunonutrition supplementation on postoperative ileus.

Eleven studies assessed postoperative ileus. The pooling results showed that the incidence of postoperative ileus in the immunonutrition group was lower than that in the control group, but the difference was not statistically significant (RR 0.73, 95% CI 0.47, 1.14; Heterogeneity: I^2^ = 0%, *p* = 0.80; Figure 5).

#### 3.3.4 Length of stay

**Figure 6:**
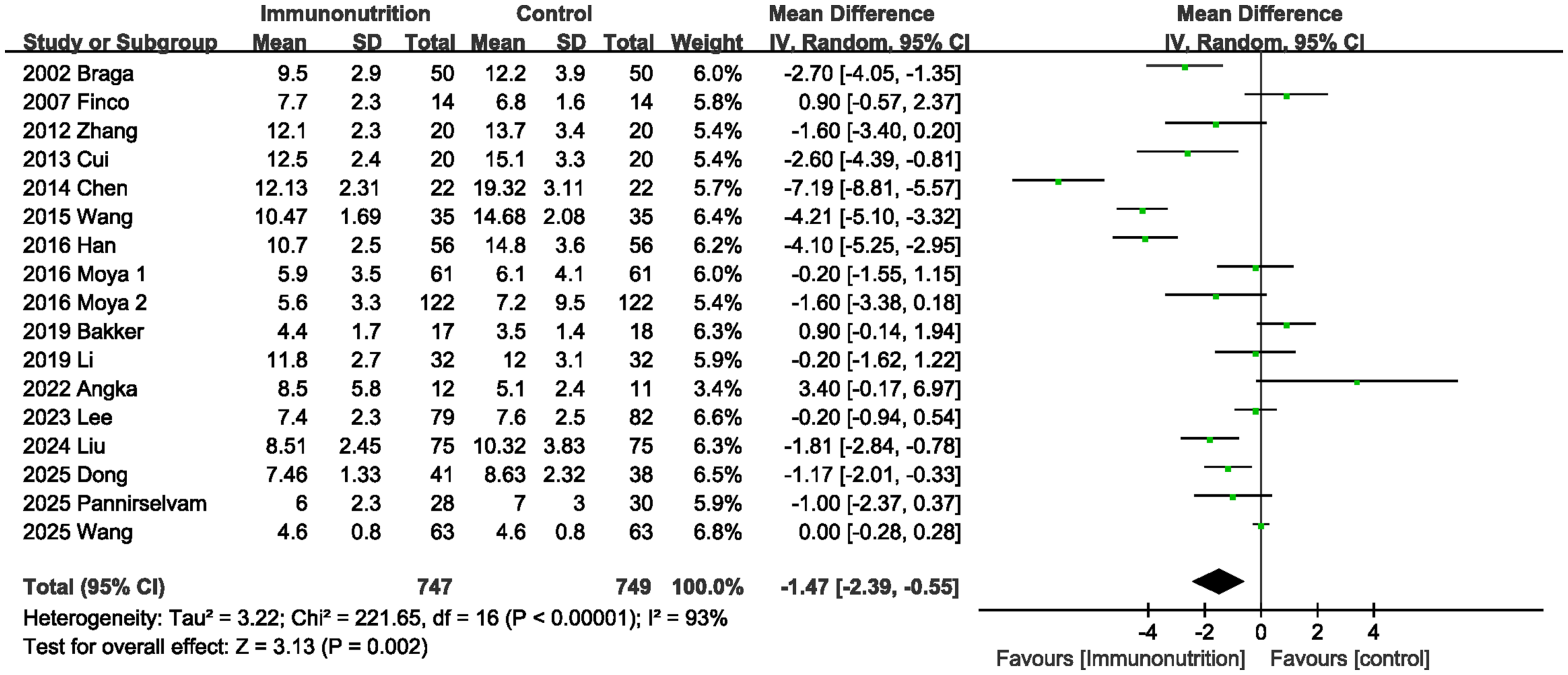
Effect of immunonutrition supplementation on length of hospital stay.

The length of hospital stay was reported in 17 studies. According to the results of this meta-analysis, supplementing with immunonutrition significantly reduced length of stay (MD, −1.47 days; 95% CI, −2.39, −0.55, *p* = 0.002; Figure 6).

#### 3.3.5 Overall postoperative complications

**Figure 7:**
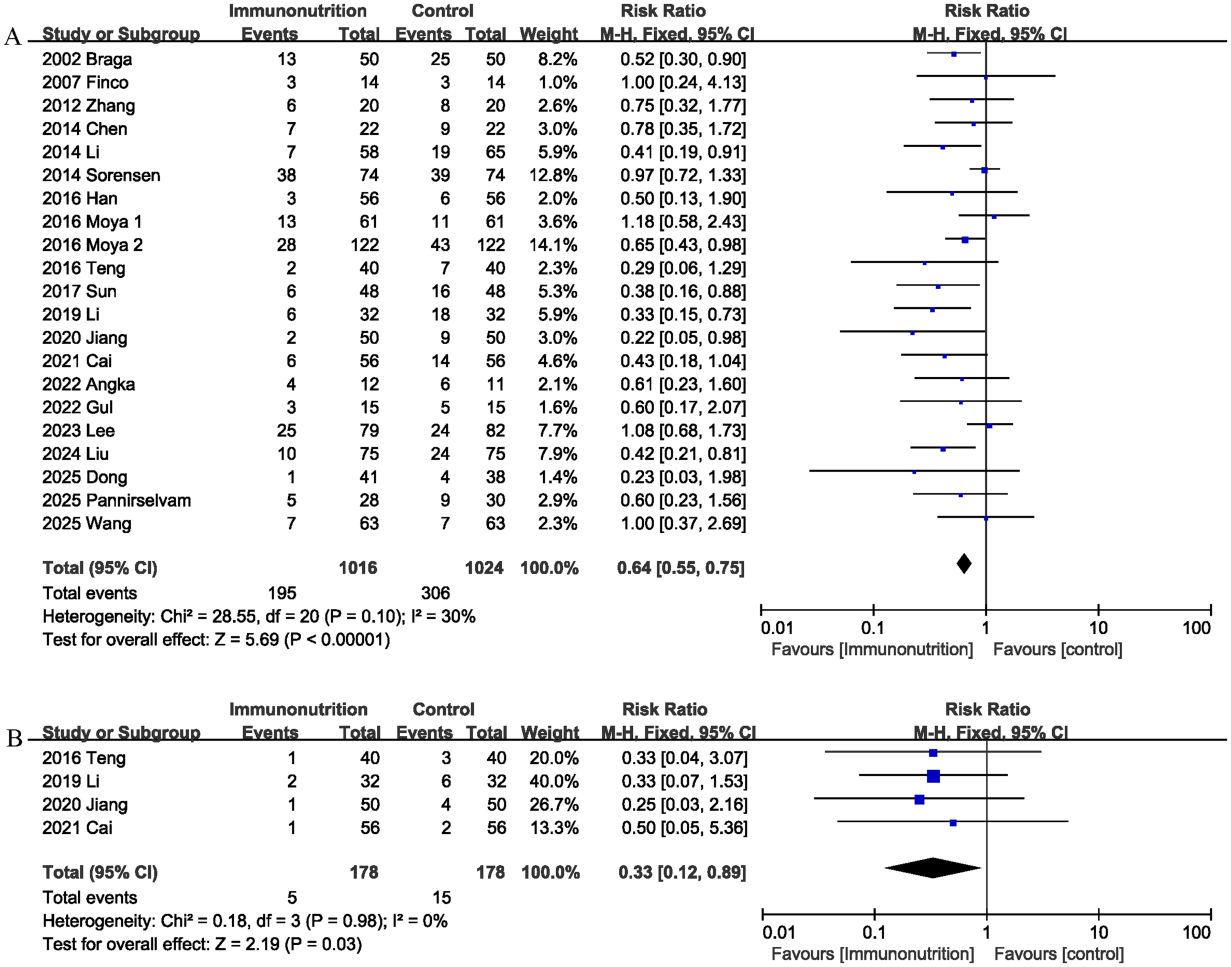
Effect of immunonutrition supplementation on **(A)** Overall postoperative complications and **(B)** Abdominal distension.

Twenty-one studies reported data on overall postoperative complications. The pooled analysis showed that the incidence of overall postoperative complications was lower in the immunonutrition group than in the control group (RR 0.64, 95% CI 0.55, 0.75; Heterogeneity: I^2^ = 30%; Figure 7A).

#### 3.3.6 Abdominal distension

Abdominal distension was reported in 4 studies, and the combined effect size suggested that immunonutrition significantly reduces postoperative abdominal distension (RR 0.33, 95% CI 0.12, 0.89, *p* = 0.03; I^2^ = 0%; Figure 7B).

### 3.4 Publication bias and sensitivity analysis

**Figure 8:**
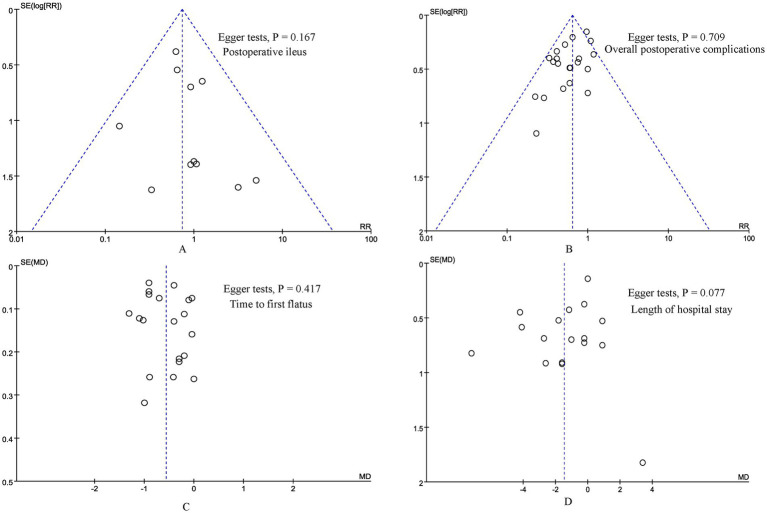
Funnel plot of **(A)** postoperative ileus, **(B)** overall postoperative complications, **(C)** time to first flatus, and **(D)** length of hospital stay.

According to the funnel plots and Egger tests (Figure 8), and no significant publication bias was observed for postoperative ileus, overall postoperative complications, time to first flatus, and length of hospital stay. Sensitivity analysis showed that no single study affected the overall effect size of the time to first flatus, time to first defecation, postoperative ileus, length of hospital stay, and overall postoperative complications. The sensitivity analysis suggested that the total effect size of abdominal distension changed significantly when the study by Li et al. (23) (RR, 0.33; 95% CI, 0.09, 1.21, *p* = 0.09; I^2^ = 0%) or Jiang et al. (21) (RR, 0.36; 95% CI, 0.12, 1.10, *p* = 0.07; I^2^ = 0%) was excluded.

## 4 Discussion

To our knowledge, this is the first meta-analysis specifically evaluating the effect of perioperative immunonutrition on postoperative bowel function recovery in patients with colorectal cancer. Unlike previous evidence syntheses that primarily focused on general postoperative complications or immune modulation, our analysis emphasizes the role of immunonutrition in promoting gastrointestinal functional recovery. Time to first flatus and time to first defecation are key indicators of postoperative gastrointestinal recovery (39). Based on pooled evidence from 28 RCTs involving 2,367 participants, immunonutrition significantly shortened the time to first flatus, time to first defecation, and postoperative length of hospital stay. Additionally, it reduced the overall incidence of postoperative complications and alleviated postoperative abdominal distension. These findings provide robust clinical evidence supporting the beneficial effect of immunonutrition on gastrointestinal recovery following colorectal cancer surgery.

### 4.1 Role of immunonutrition

Postoperative ileus remains a common complication and one of the primary contributors to delayed hospital discharge after colorectal cancer surgery (40). Its etiology is multifactorial and closely associated with surgical trauma–induced inflammatory responses, autonomic nervous dysfunction, opioid use, and electrolyte imbalances (41–43). Surgical manipulation activates the sympathetic nervous system, thereby suppressing gastrointestinal motility. Neuropeptides such as substance P and nitric oxide released from the enteric nervous system may further prolong postoperative bowel dysfunction (6). Moreover, the inflammatory cascade triggered by surgery results in the release of interleukin-1, monocyte chemoattractant protein-1, intercellular adhesion molecule-1, and interleukin-6, which can damage intestinal smooth muscle cells and impede bowel recovery (43). Opioids administered perioperatively also slow gastrointestinal transit via *μ*-opioid receptor activation on interstitial cells of Cajal (6).

Immunonutrition has been widely implemented in patients with gastrointestinal malignancies to improve nutritional and immune status, prevent postoperative infections, and reduce morbidity (44–46). Common immunonutrients include glutamine, omega-3 polyunsaturated fatty acids, and arginine. Glutamine is a conditionally essential amino acid that may become depleted under stress or infection (47). It serves as a major metabolic substrate for enterocytes and immune cells, helping to maintain intestinal integrity and modulate immune homeostasis (44). Glutamine depletion has been associated with increased intestinal permeability, impaired immune function, and higher risks of infection (39). Evidence suggests that glutamine supplementation prevents mucosal atrophy, protects the intestinal barrier, and mitigates therapy-induced gastrointestinal toxicity such as diarrhea and mucositis (48, 49). In animal studies, perioperative glutamine also enhanced postoperative gastrointestinal recovery after abdominal surgery (50).

Arginine plays a critical role in collagen synthesis and wound healing and supports immune function by promoting T-cell activation (44). It may suppress the production of proinflammatory cytokines such as tumor necrosis factor-*α* (TNF-α) and interleukin-6 (IL-6) (44, 47). As a precursor of nitric oxide, arginine improves microvascular perfusion and may counteract hypoxia-related gut dysmotility and inflammation-associated microcirculatory dysfunction during surgery (47). Omega-3 fatty acids exert immunomodulatory and anti-inflammatory effects (51). Meta-analytic evidence indicates that omega-3 supplementation significantly reduces circulating TNF-α and IL-6 levels (52). Omega-3 fatty acids may also beneficially alter gut microbiota composition, reduce systemic inflammation, and promote postoperative recovery (53). Collectively, these mechanisms support the observed clinical improvements. Our meta-analysis confirms these potential benefits by demonstrating significant reductions in time to first flatus, time to first defecation, abdominal distension, and hospital stay, consistent with previously published retrospective studies (39, 54).

### 4.2 Clinical implications

Postoperative complications not only increase healthcare expenditure but may also negatively affect long-term outcomes. Moreover, postoperative complications are closely associated with delayed hospital discharge (55, 56). Huang et al. reported that postoperative wound infection after rectal cancer surgery prolongs hospitalization and reduces overall survival (55). Consistent with previous meta-analyses, our findings indicate that immunonutrition lowers postoperative complication rates (44, 57). This reduction likely contributes to the shorter hospital stay observed in the immunonutrition group. Several studies (5, 58) have shown that delayed postoperative gastrointestinal recovery and prolonged hospital stays are associated with poorer clinical outcomes. Supplementation with immunonutrition reduces the time to gastrointestinal recovery by approximately 0.5 days and shortens hospital stays by about 1.5 days. Although these absolute reductions may seem modest, with advances in surgical techniques and equipment, even small improvements in recovery time can be clinically significant, especially when applied to patients undergoing colorectal surgery, particularly those following enhanced recovery after surgery (ERAS) protocols.

### 4.3 Strengths of the study

This study has several strengths. First, we employed a comprehensive and highly specific search strategy covering major biomedical databases, minimizing the risk of missing relevant studies. Second, we included only RCTs, ensuring a high level of evidence, reduced selection bias, and improved baseline comparability between intervention and control groups.

### 4.4 Limitations

Our study has the following limitations. First, although 28 RCTs were included, some studies had relatively small sample sizes, which may reduce statistical power at the individual trial level. Secondly, substantial heterogeneity was observed in some outcome measures. Heterogeneity in immunonutrition formulas, timing of administration (preoperative, postoperative, or perioperative), and duration of supplementation contributed to clinical variability. Future research should determine the optimal timing, regimen, and dosage of immunonutrition. In addition, neoadjuvant chemoradiotherapy is often associated with gastrointestinal adverse effects and may influence surgical outcomes. The inclusion of heterogeneous patient populations (with or without neoadjuvant treatment) may represent an important source of heterogeneity in the present analysis. Because several included studies enrolled mixed populations and did not report outcomes separately, subgroup analysis according to neoadjuvant treatment status could not be performed. Future studies should further explore the effects of immunonutrition in specific patient populations, such as those receiving neoadjuvant therapy. Finally, most included trials were single-center studies, which may limit generalizability across different geographic or institutional settings.

### 4.5 Future directions for research

Elderly patients, individuals with malnutrition, and patients with preoperative comorbidities are at higher risk of delayed postoperative gastrointestinal recovery. Future RCTs should specifically evaluate the effects of immunonutrition in these clinically relevant subgroups, such as elderly patients, malnourished individuals, and patients with preoperative comorbidities. Additionally, although postoperative ileus is a common complication, there is currently no universally accepted definition or standardized diagnostic criteria. We reviewed the studies included in our meta-analysis, and none provided detailed definitions of postoperative ileus. Variations in definitions may be a significant source of heterogeneity in this study, which could result in differences in outcome assessment and influence the interpretation of pooled results. Future research should adopt clear and standardized definitions to further evaluate the impact of immunonutrition on postoperative ileus.

In conclusion, evidence from 28 RCTs suggests that perioperative immunonutrition significantly shortens the time to first flatus and time to first defecation, reduces overall postoperative complications, and shortens hospital stay in patients undergoing colorectal cancer surgery. However, immunonutrition supplementation did not reduce the incidence of postoperative ileus. Given the high heterogeneity, the effects of immunonutrition on postoperative gastrointestinal recovery should be interpreted with caution. Future multicenter RCTs using standardized postoperative ileus definitions, uniform immunonutrition formulations, and supplementation protocols are necessary to validate these findings and further clarify its role in postoperative gastrointestinal functional recovery.

## Data Availability

The original contributions presented in the study are included in the article/supplementary material, further inquiries can be directed to the corresponding author.
